# Current landscape of climate change adaptation and health preparedness among indigenous populations in Southeast Asia

**DOI:** 10.1002/puh2.129

**Published:** 2023-10-12

**Authors:** Sheena Ramazanu, Lowilius Wiyono, Hammoda Abu‐Odah, Rey G. Comabig, Shuaibu Saidu Musa, Jemilah Mahmood, Yong Zhin Goh, Nurul Amanina Binte Hussain, Siddharthen Rajasegaran, Thakshayeni Skanthakumar, Adriana Viola Miranda

**Affiliations:** ^1^ Leadership Institute for Global Health Transformation Saw Swee Hock School of Public Health National University of Singapore Singapore Singapore; ^2^ Abdul Aziz General Hospital Singkawang Indonesia; ^3^ School of Nursing The Hong Kong Polytechnic University Kowloon Hong Kong; ^4^ Institute of Arts and Sciences Southern Leyte State University, Sogod Southern Leyte Philippines; ^5^ Department of Nursing Science Ahmadu Bello University Zaria Nigeria; ^6^ Sunway Centre for Planetary Health Sunway University Petaling Jaya Selangor Malaysia; ^7^ Institute of Tropical Medicine and International Health Charité – Universitätsmedizin Berlin Berlin Germany; ^8^ Singapore Indian Development Association (SINDA) Singapore Singapore; ^9^ Infectious Disease Research and Training Office National Centre for Infectious Diseases Singapore Singapore; ^10^ Global Health Focus Asia Bandung Indonesia

**Keywords:** climate change, indigenous populations, strategies, vulnerability

## Abstract

Human‐induced climate change poses a pervasive threat to the world. Human activities, such as deforestation, farming livestock, and burning fossil fuels, are key drivers of climate change. Like other regions, the Southeast Asian region is greatly impacted by climate change. The article focuses on examining the current landscape of climate change adaptation and health preparedness among indigenous populations in Southeast Asia (SEA). Climate change will affect the indigenous populations disproportionately. Over the years, indigenous people living in SEA have faced increasing challenges. For instance, air pollution resulting from forest fires causes respiratory conditions, skin irritations, and other significant health risks. The article also highlights climate change–related health system preparedness in ASEAN and indigenous strategies in navigating climate change adaption. As the saying goes, “actions speak louder than words.” To develop sustainable regional climate change adaptation strategies, representation and voices of indigenous peoples matter. At ASEAN level, although the ASEAN Working Group on Climate Change was convened to develop policies and coordinate action plans among its member states, it is now key to include and learn from the instrumental strategies of indigenous communities in conserving, protecting, and restoring forests. Beyond acknowledging the efforts of indigenous communities on paper, it is now time to translate scientific knowledge into practical actions. It is necessary for us to value and recognize indigenous peoples, particularly in SEA as valued agents in co‐creating sustainable solutions for climate agenda. Centering indigenous peoples’ knowledge in climate adaptation is crucial for strengthening collective resilience in climate action strategies. Conclusively, the article advocates for the prioritization of indigenous communities’ leadership efforts in ASEAN‐wide climate action initiatives and climate action policy.

## INTRODUCTION

Climate change has become a global disaster due to global warming driven by human activities. Southeast Asia (SEA) like other regions is affected by climate change. The key drivers of climate change globally and in SEA include the burning of fossil fuels, deforestation, and farming livestock. SEA is also one of the most affected regions due to climate change, particularly sea‐level rise with its long coastline, growing economic activities, abundant low‐lying areas, and their reliance on agriculture. SEA consists of eleven countries: Vietnam, Indonesia, Malaysia, Laos, Myanmar, Singapore, Brunei, Philippines, Cambodia, Thailand, and Timor‐Leste (observer of ASEAN). By 2030, rising sea levels alone could result in US$724 billion expenditure in big cities. Areas of Jakarta, Indonesia could be totally submerged, whereas Bangkok, Thailand will be impacted hard with almost 10 million people being affected from coastal flooding, risking 96% of country's gross domestic product to damages and losses [1]. In Brunei Darussalam, heat‐related mortality among older adults (aged 65 years and above) is predicted to significantly increase from zero deaths per 100,000 in 1990–51 deaths per 100,000 by 2080 [2]. Similarly, in Cambodia, under a high emission scenario, heat‐related mortality is expected to increase up to 56 deaths per 100,000 by 2080 in older adults [3]. As a result of climate change, temperature is expected to rise with delayed monsoon season and drier weather condition in Indonesia, leading to increase the incidence of malaria, dengue, and cholera cases [4].

These climate changes will affect the indigenous populations disproportionately. Indigenous populations are heterogenous group of people with distinct cultural and social communities who share collective ancestral ties to the lands. SEA is home to 150 million indigenous peoples across various tribes or ethnicities scattered across the region, such as the Dayak people in Malaysia and Indonesia or the Ilongot in the Philippines. They are heavily affected by the adverse impacts of climate change, such as depleting water resources and fragmentation of forest [5]. Indigenous communities are socially, culturally, and spiritually connected to their lands for basic resources and livelihoods [6]. With rising global temperatures, the usual preparation and storage of traditional foods could be affected, increasing health risks such as gastroenteritis and food‐borne botulism among indigenous populations [7]. In recent years, air pollution from forest fires poses a significant risk to indigenous communities’ health, causing respiratory conditions, eye, and skin irritations [4].

Furthermore, indigenous people are at the forefront of experiencing various mental health impacts of climate change. The available evidence suggests that the adverse effects of climate change are closely intertwined with powerful emotional responses, such as depression and anxiety [8]. Both qualitative and quantitative research studies conducted in countries like Canada, Australia, and Russia have shown that continuous environmental degradation is associated with reduced mental well‐being among indigenous communities. These negative impacts manifest in various forms, including feelings of sadness, worry, fear, diminished self‐esteem, and emotional distress [8]. This commentary aims to scrutinize the current landscape of climate change adaptation and health preparedness among indigenous populations in SEA.

## CLIMATE CHANGE–RELATED HEALTH SYSTEM PREPAREDNESS IN ASEAN

Each country in SEA has included health as a key sector of climate change adaptation and mitigation plans. ASEAN Action Plan on Joint Response to Climate Change was initiated in 2012 to leverage opportunities such as capacity building of climate‐friendly technology and knowledge transfer within the region [9]. ASEAN Socio‐Cultural Community Blueprint 2025 recommended strengthened social protection for communities living in at‐risk areas [10]. In 2009, the ASEAN Working Group on Climate Change (AWGCC) was launched to examine issues related to changing climate, develop policy recommendations, and coordinate responses among member states. Each year, AWGCC meets to discuss and report on action plans and strategize directions on strengthening science and policy linkages [11]. These documents unfortunately did not address indigenous people's fight against climate change. Representation of indigenous peoples’ voices especially in the Southeast Asian region is pivotal to develop to regional climate change adaptation strategies.

## CHANGING CLIMATE AND INDIGENOUS STRATEGIES

Climate change hits vulnerable populations such as marginalized communities (persons with disabilities, indigenous Peoples, ethnic minorities, and displaced persons) the hardest [12]. During the pandemic, the indigenous communities faced even more threats. Indigenous villages in Northern Thailand battled with combined threats of forest fires and the COVID‐19 pandemic [13]. Slash and burn practices related to agribusiness were likely to have caused the forest fires, contributing to unhealthy air levels amid the COVID‐19 pandemic. Air pollution has been shown to increase the health impact and risks of COVID‐19. In addition to that, climate change causes disruptions to food and agricultural chains. The COVID‐19 pandemic exposed health inequities and vulnerabilities of indigenous communities, stemming from lack of access to national health systems, food security, sanitation facilities, and water [14].

Due to climate change–related losses of environmental knowledge, cultural identity, destruction of ecosystems, and species, indigenous communities are expected to experience “ecological grief” [15]. In the Southeast Asian context, indigenous solutions to tackling this crisis include embracing traditional underpinnings of collective resilience and community building. In Malaysia, Sarawak, indigenous communities (Lun Bawang, Saban, and Penan) utilize sky‐color changes indigenous forecasts and animal migration to recognize climate changes. These examples illustrate the potential of indigenous knowledge and climate adaptation techniques to be embedded within contemporary climate‐risk assessments [16]. Spiritual rituals are performed by some communities to draw spiritual strength and declare commitment to support one another during unprecedented times. In the Philippines, indigenous peoples of the Cordillera practice “binnadang” where communities unite to share food and support those in need [17]. Indigenous communities can bring fresh perspectives, skills, and traditional ecological knowledge on strategies to survive the effects of changing climate. Recognizing these strengths, the Southeast Asian countries have included the identification and inclusion of climate change–related indigenous knowledge in their adaptation actions, albeit with a somewhat low priority score (an average of 3 out of 10) [18].

## RECOMMENDATIONS

The key recommendation would be to recognize that the indigenous people are exceptionally vulnerable and affected by climate change; hence need special measures to address their needs. ASEAN‐wide climate action initiatives should prioritize indigenous communities’ leadership, empowerment, and collaboration in climate action policy. Previous efforts have included community‐based initiatives, such as early warning systems [18], disaster response plans, and sustainable livelihood projects. However, there are still significant challenges that need to be addressed to improve the health preparedness of indigenous populations in SEA.

Cultural and world views of indigenous populations should be respected and built upon. Wisdom, values, and knowledge of indigenous peoples have been largely silenced in global policy, climate science, and advocacy. A key challenge would be bringing people from diverse communities and cultures together to engage in climate action conversations [19]. Building trust and cultivating relationships are critical components to ensure that engagements take place in an equitable manner, empowering people from different walks of life to work together in solving a common global issue. In the Southeast Asian context, relevant programs can be curated to bring indigenous communities together, which serves as an opportunity to learn and coordinate ways of intercultural collaborative work on managing climate crisis. Additionally, the insufficiency of data on the impact of climate change on indigenous communities, particularly in SEA, represents a critical challenge. Given that this region is among the most vulnerable to the effects of climate change, it is imperative to enhance the accessibility and accuracy of data to gain a better comprehension of the contextualized strategies that indigenous peoples employ to maintain their health amidst the changing climate.

Finally, actions speak louder than words. The acknowledgment of indigenous communities would not just be on paper, but also in the real‐world actions. Some strategies would be initiating periodic policy dialogues and working groups to ensure indigenous communities’ voices are heard, in ASEAN and in each member state. Knowledge transfer between member states about the opportunities and challenges of including indigenous communities in the climate change–related health preparedness is essential.

## CONCLUSION

It is essential that indigenous peoples are valued as agents of change to strategize what works best for our planet in mitigating climate crisis. Tapping on indigenous knowledge is necessary to co‐create enriched solutions for sustainable climate agenda in SEA.

## CONFLICT OF INTEREST STATEMENT

Sheena Ramazanu, Shuaibu Saidu Musa, and Adriana Viola Miranda are Editorial Board members of Public Health Challenges and co‐authors of this article. To minimize bias, they were excluded from all editorial decision‐making related to the acceptance of this article for publication.

## FUNDING INFORMATION

There is no funding in the development for this article.
